# Purification and Characterization of a Cold-Adapted Lipase from *Oceanobacillus* Strain PT-11

**DOI:** 10.1371/journal.pone.0101343

**Published:** 2014-07-01

**Authors:** Tian Jiewei, Lei Zuchao, Qiu Peng, Wang Lei, Tian Yongqiang

**Affiliations:** 1 Key laboratory of Leather Chemistry and engineering, College of Light Industry, Textile and Food Engineering, Sichuan University, Chengdu, China; 2 Department of Pharmaceutical and Biological Engineering, College of Chemical Engineering, Sichuan University, Chengdu, Sichuan, China; Queen's University Belfast, United Kingdom

## Abstract

We isolated a moderately halophilic lipase-producing bacterium from the saline soil. Based on the morphological, physiological, chemotaxonomic and phylogenetic analysis, the isolate PT-11 was postulated to be a novel species identified as *Oceanobacillus rekensis* PT-11. The lipase was purified 2.50-fold by Q-Sepharose FF and SP-Sepharose FF chromatography and its molecular mass was estimated to be 23.5 kDa by SDS-PAGE. It was highly active over the broad temperature ranging from 10 to 35°C and showed up to 80% of the maximum activity at 10°C indicating the lipase to be a typical cold-adapted enzyme. The enzyme activity was slightly enhanced by Na^+^, Li^+^ and K^+^. Incubation with detergents, such as Tween-20 and Tween-80, slightly inhibited the enzyme activity; while Triton X-100decreased the enzyme activity. The enzyme was fairly stable in the presence of long-chain alcohols but was highly denatured in hydrophilic solvents such as acetone or short-chain alcohols (C1–C3).

## Introduction

Extremophilic organisms play a surprisingly important role in the scientific community because of the molecular adaptation they underwent during evolution and for their biotechnological potential [1], [2], [3]. The extremophiles face hyperhaline, organic, hypertonic or extreme temperature enviroments; therefore the extremophiles have to change properties of their macromolecules in order to maintain normal physiological activity in extreme conditions. These macromolecules display clearly distinguished special features such as hyperhaline, organic, hypertonic or extreme temperature tolerance [4], [5].

Lipases represent a class of enzyme that catalyzes the cleavage and formation of ester bonds. Many of them have wide substrate tolerance, high stereospecificity toward chemicals, and high stability in organic solvents [6], [7]. Thus, they are very interesting biocatalysts for industrial purposes such as detergency, flavour production, paper recycling, chemical synthesis, resolution of racemic mixtures [8], [9], [10].

Since the industrial processes are usually carried out under extreme conditions, it is very important to isolate the lipases which keep relatively high activity under diverse conditions [4]. For this reason, the lipases isolated from extremophiles constitute a perfect substitute for industrial process [11]. Although a large number of lipases from the non-halophilic bacteria have been reported, only a very few lipases from the moderately halophiles or extremely halophiles have been studied so far, and there are almost no reports about cold-adapted lipases [12].

In our study, we isolated a lipase-producing bacterium, of the genus *Oceanobacillus*, strain PT-11, from the saline soil Shache, Xinjiang Province, northwest of China, and described purification of its intracellular lipase and characterization of the lipase with respect to the biochemical properties.

## Materials and Methods

### Microorganisms and media

The soil sample was collected from Shache county (77°14.908′E, 38°25.049′N), west of Tarim Basin in Xinjiang Province, northwest of China. Saline soil in Shache is the typical type of saline soil in Xinjiang, where the main ions are chlorine and sulphate. The typical inland arid climate and special geographical conditions make salt accumulate on the surface of the soil profile. pH value of saline soil at sampling sites varied from 7.41–7.97. The soil samples were collected at a depth of 10–30 cm at each sampling site and stored in 50-ml sterile Falcon centrifuge tubes (Shanghai Sangon, China). Our study didn't require specific permissions, because the sample did not involve endangered or protected species. The study wasn't related to any animal experiments. This isolate was grown in a saline medium (TSB-7.5) with a total NaCl concentration of 10% (w/v) supplemented with 1.5% (w/v) tryptone, 0.5% (w/v)soya peptone and the pH of the medium was adjusted to 7.5. The isolate was cultivated at 37°C in an orbital shaker at 200 rpm.

### Identification of strain PT-11

The isolate PT-11 was identified by morphological, physiological, chemotaxonomic and molecular assays. The shape and color of the colony, Gram reaction, motility, urease and oxidase activities, nitrate reduction, catalase, Voges-Proskauer and methyl red, Tween-80 hydrolysis tests were performed according to the method described previously [13]. Scanning electron microscopy (SEM) (JSM-7500F, JEOL) was performed at the Analytical and Testing Center Sichuan University using cells from the isolate PT-11 grown in TSB medium for 72 h at 30°C. Tests for utilization of different substrates were performed by BIOLOG-GNIII system and other enzymatic activities were assayed by using API ZYM strips according to the manufacturer's instructions with 3% (w/v) NaCl.

To confirm the identity of the isolate, total genomic DNA of isolated microorganism was extracted as previously described [14] and the 16S rDNA gene was amplified using the universal primers 27F(5′-CCGTCGACGAGCTCAGAGTTTGATCCTGGCTCAG-3′) and 1492R(5′-CCCGGGTACCAAGCTTAAGGAGGTGATCCAGCCGCA-3′). PCR was carried out in a total volume of 50 µl containing PCR buffer with 7.5 µM MgCl_2_, 200 µM dNTPs, Taq DNA polymerase (1.0 U), 0.5 µM each primer, and DNA template (∼80 ng). The amplification procedure was as follows: initial denaturation at 95°C for 4 min, and 30 cycles of denaturation at 95°C for 45 s, annealing at 70°C for 45 s, and extension at 72°C for 1 min, with final extension at 72°C for 10 min. The PCR products were examined on 1% agarose gel. For sequencing, the amplified PCR products were purified using a Sangon DNA Fragment Purification Kit and sequenced using an ABI 3730 automated sequencer at Shanghai Sangon Biotech (Shanghai, China). The sequences were aligned with Clustal W [15]. Phylogenetic tree (neighbor-joining) was constructed using MEGA 4.0 from dissimilar distances and pairwise comparisons with the Kimura 2-parameter model [16].

### Nucleotide sequence accession number

The 16S rDNA sequence of isolate PT-11 was submitted to the NCBI under accession number HQ620695.

### Lipase activity assay

This assay was performed by measuring the increase in the absorbance at 410 nm produced by the release of *p*-nitrophenol in the hydrolysis of 0.4 mM *p*-nitrophenyl acetate (*p*-NPA) in 50 mM Tris-HCl buffer at pH 8.5 and 30°C. To initialize the reaction, 0.1 ml of lipase solution or suspension was added to 4.9 ml of substrate solution. The assay was slightly modified by replacement of the chromogenic substrate [17]. One lipase unit was defined as the amount of enzyme required to liberate 1 µmol of *p*-nitrophenol per minute.

### Purification of enzyme

All purification steps were carried out at room temperature, unless otherwise specified.

#### Step.1 Cell disruption

Cells from a 72 h old culture of isolate PT-11 growing at 37°C {modified TSB medium (1%(v/v) olive oil, 10% (w/v)NaCl, 1.5% (w/v) tryptone and 0.5% (w/v)soya peptone pH 7.5)}were harvested by centrifugation at 10000×g for 10 min at 4°C. The culture supernatant was removed and the pellet was washed twice in 50 mM Tris-HCl buffer (pH 8.5). The cells were disrupted by ultrasonic treatment (Labsonic, Braun Biotech International) for 30 min and the cell debris was removed by centrifugation at 10000×g for 10 min at 4°C. The resulting supernatant was kept as the intracellular fraction and was stored at −20°C until use.

#### Step.2 Anion-exchange chromatography

The samples resulting from cell disruption were applied onto a Q-sepharose FF column previously equilibrated with 50 mM Tris-HCl buffer (pH 8.5). The column was washed with the same buffer, and eluted with 50 mM Tris-HCl buffer (pH 8.5) containing 1 M NaCl, then followed with a linear gradient of NaCl (0–1 M) of the same buffer. The activity was assayed and the active fractions were pooled, and then dialyzed against 20 mM phosphate buffer (pH 6.0).

#### Step.3 SP-sepharose FF chromatography

The active fractions were applied onto SP-sepharose FF column previously equilibrated with 20 mM phosphate buffer (pH 6.0). The column was washed with the same buffer, and eluted with 20 mM phosphate buffer (pH 6.0) containing 1 M NaCl, then followed with a linear gradient of NaCl (0–1 M) of the same buffer. The active fractions showing lipase activity were pooled.

### Determination of molecular mass

The molecular mass of the denatured protein was determined by sodium dodecyl sulfate-polyacrylamide gel electrophoresis (SDS-PAGE) [18]. SDS-PAGE was performed with a 15% polyacrylamide gel on a vertical mini gel apparatus (Bio-RAD) at 200 V for 40 min.

### Characterization of lipase

#### Effect of pH on enzyme activity and stability

For the optimum pH determination, the activity was determined by assay at 30°C in a pH range of 6.0–10 using the different buffers: phosphate buffer for pH 6.0, 6.5, 7.0, 7.5, 8.0; Tris–HCl buffer for pH 8.0, 8.5, 9.0; borate saline buffer for pH 9.0, 9.5, 10.0. The activity was measured after 10 minutes by assay. For the pH stability, the purified lipase was incubated at 30°C in a pH range of 6.0–10.0 after 30 min using the different buffers: phosphate buffer for pH 6.0–8.0; Tris–HCl buffer for pH 8.0–9.0; borate saline buffer for pH 9.0–10.0 and the residual activity was measured after 10 minutes by assay.

#### Effect of temperature on enzyme activity and stability

For the optimum temperature determination, the activity was measured at the different temperatures (10–90°C) at pH 8.5 in 50 mM Tris-HCl buffers. The activity was measured after 10 minutes by assay. For thermostability, the purified lipase was incubated at 10–50°C for up to 30 min in 50 mM Tris-HCl, pH 8.5 and the residual activity was measured after 10 minutes by assay.

#### Effect of various metal ions and detergents on lipase activity

The effect of various additives on lipase activity was investigated by pre-incubating for 3 min at 30°C, containing 1 mM of CaCl_2_, CuCl_2_, KCl, NaCl, LiCl and 0.2% of Tween-20, Tween-80 and Triton X-100.

#### Effect of organic solvents on lipase activity

The effect of organic solvents [methanol, ethanol, acetonitrile, glycerol and dimethyl sulfoxide (DMSO)] on the activity of lipase was determined. Equal volume of the organic solvents and the purified enzyme was incubated separately at 30°C for 10 min.

### Protein estimation

Protein concentration was determined by the Bradford method using bovine serum albumin as standard [19].

## Results

### Identification of PT-11

**Figure 1:**
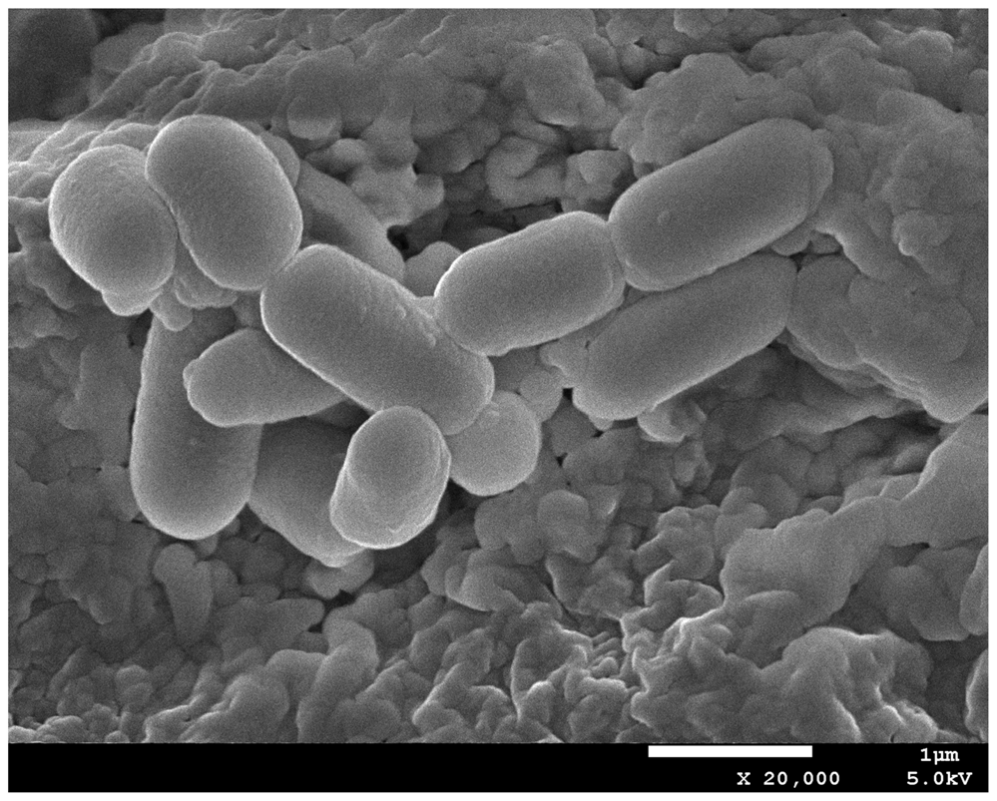
SEM micrographs of *Oceanobacillus rekensis* PT-11.

Based on the morphological and physiological tests, the bacterial strain PT-11, isolated from a saline soil in Shache, was confirmed as a Gram-positive, motile, irregular short rod, and aerobic spore-form (Fig.1). Colonies are circular, smooth and creamy. Growth occurs with 5–20% (w/v) NaCl (optimum 12%), at pH 6.5–10.0 (optimum pH 8.0) and at 15–40°C (optimum37°C). Oxidase, catalase, hydrolysis of Tween 80, gelatinase and nitrate reduction tests were positive, while urease, Voges–Proskauer, H2S production and methyl red tests were negative.

Tests for the utilization of different carbon sources were performed by the BIOLOG-GNIII system and the result was shown in table S1. The enzymatic activities were assayed by using API ZYM strips according to the manufacturer's instructions and the result was shown in table S2.

The amino acid in the cell-wall peptidoglycan of the isolate PT-11 was meso-diaminopimelic acid and the predominant menaquinone was MK-7. The major cellular fatty acids were anteiso-C15:0, iso-C15:0, iso-C14:0, iso-C16:0 and anteiso-C17:0. The polar lipid profile consisted of phosphatidyl glycerol. The G+C content of the genomic DNA was 36.7 mol%, in the range 32–69 mol% accepted for species of genus *Oceanobacillus*. The chemotaxonomic properties were all typical characteristics of the genus *Oceanobacillus*.

**Figure 2:**
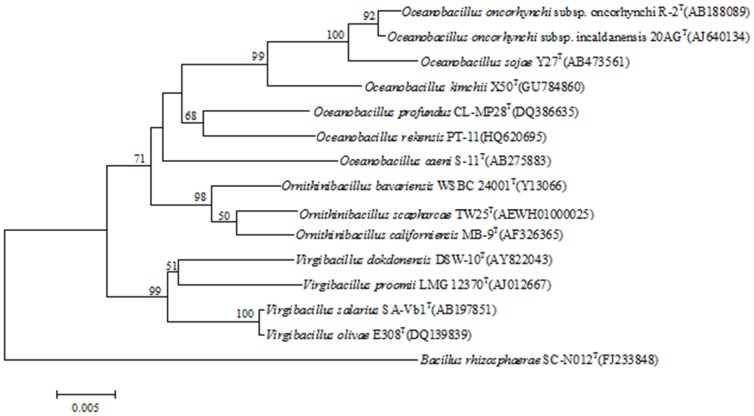
Phylogenetic tree based on 16S rDNA sequences, showing the relationship of strain PT-11 to other members of the genus *Oceanobacillus*. Accession numbers of the sequences used in this study are shown in parentheses after the strain designation. Numbers at nodes are percentage bootstrap values based on 1,000 replications; only values greater than 50% are shown. Bar 0.0005 substitutions per nucleotide position.

In this work, the complete 16S rDNA sequence of isolate PT-11 was obtained. Based on 16S rDNA analysis the phylogenetic position of isolate PT-11 was determined to be within the strain *Oceanobacillus rekensis*.sp PT-11 (AccessionNo. HQ620695) (Fig.2). The close relative, *Oceanobacillus profundus* CL-MP28T (AccessionNo. DQ386635) showed 97.267% similarity to this strain.

To confirm whether or not strains *Oceanobacillus profundus* CL-MP28T and the PT-11 isolate truly constituted a single novel species of the genus *Oceanobacillus*, DNA–DNA hybridizations were performed. DNA–DNA hybridizations were performed with strains *Oceanobacillus profundus* CL-MP28T and the PT-11 isolate using the microplate method [20]. Two hybridizations were performed and the DNA–DNA relatedness values respectively were 45.8% (*Oceanobacillus profundus* CL-MP28T×PT-11) and 51.5% (PT-11×*Oceanobacillus profundus* CL-MP28T). As levels of 70% DNA–DNA relatedness are generally accepted as the limit for species definition [21], the DNA–DNA hybridization results confirmed that the strain PT-11 represented a single novel species of the genus *Oceanobacillus*.

Based on morphological, physiological, chemotaxonomic and phylogenetic analysis, the isolate PT-11 was determined to be within the strain *Oceanobacillus rekensis*. PT-11.

### Purification of the enzyme

**Figure 3:**
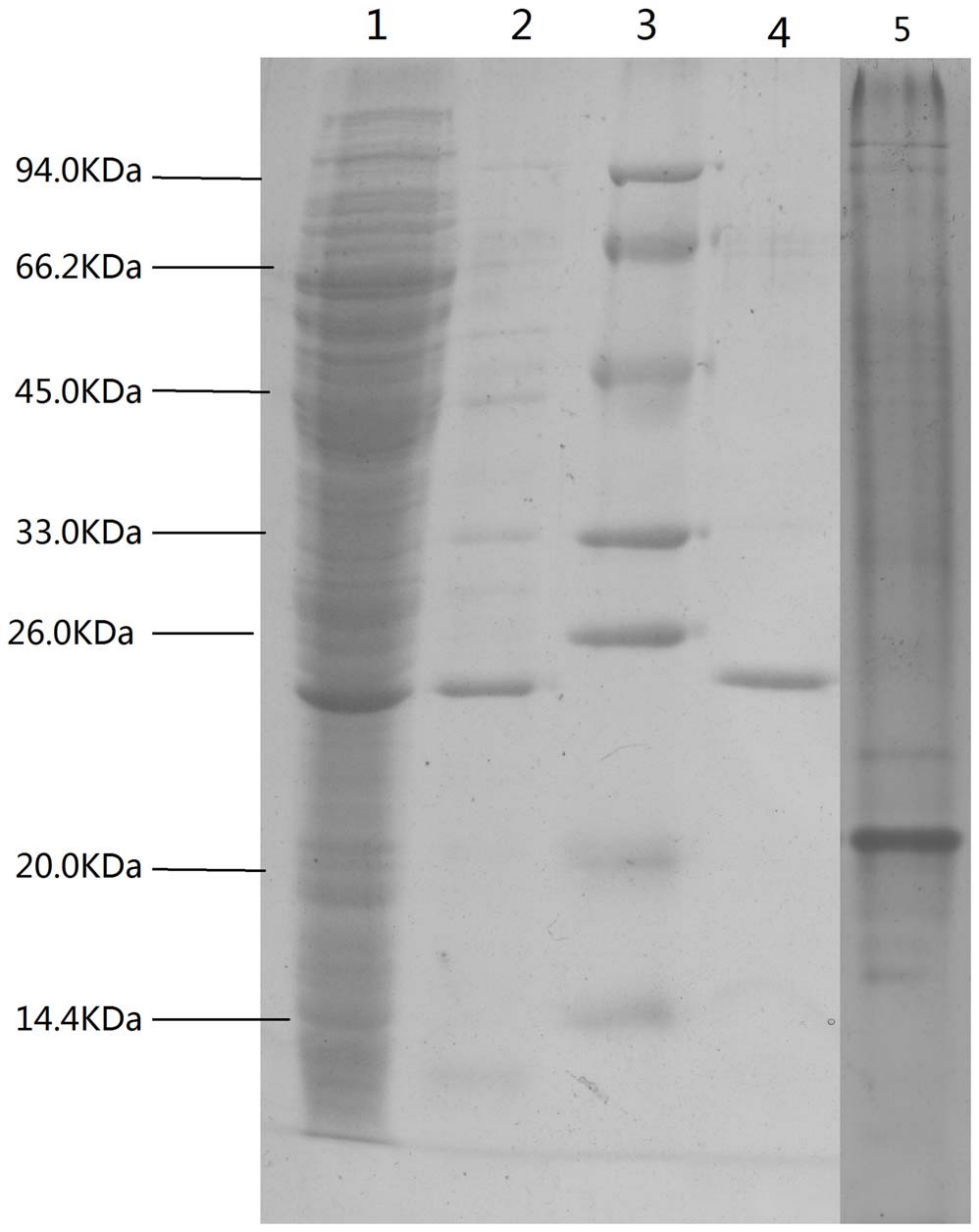
SDS-PAGE of lipase. (20 ul sample) (1) Total after cell disruption. (2) Purified by anion-exchange chromatography. (3) SDS-PAGE molecular weight standards. (4) Purified by Cation-exchange chromatography. (5) Native-PAGE.

The lipase from *Oceanobacillus rekensis* PT-11 was purified 2.50 fold in 3 steps and the specific activity was 65.55 U/mg-protein with a yield of 14.80% (Table 1).The specific activity of the enzyme increased 2.33-fold by anion-exchange chromatography on Q-sepharose FF. The next step, cation-exchange chromatography on SP-sepharose FF, was effective for the purification of the enzyme with 2.50-fold increase in the specific activity. The molecular mass of enzyme was 23.5 KDa approximately (Fig.3).

**Table 1:** Purification of the lipase.

Purification of lipase	VoL(ml)	Total protein(mg)	Total activity(U)	Specific activity (U/mg)	Purification (fold)	Yield (%)
Cell disruption	90	163.72	4295.55	26.23	1	100
Q-Sepharose FF	40	15.65	954.21	61.01	2.33	22.21
SP-Sepharose FF	30	9.70	635.73	65.55	2.50	14.80

### Characterization of lipase

#### Effect of pH on enzyme activity and stability

**Figure 4:**
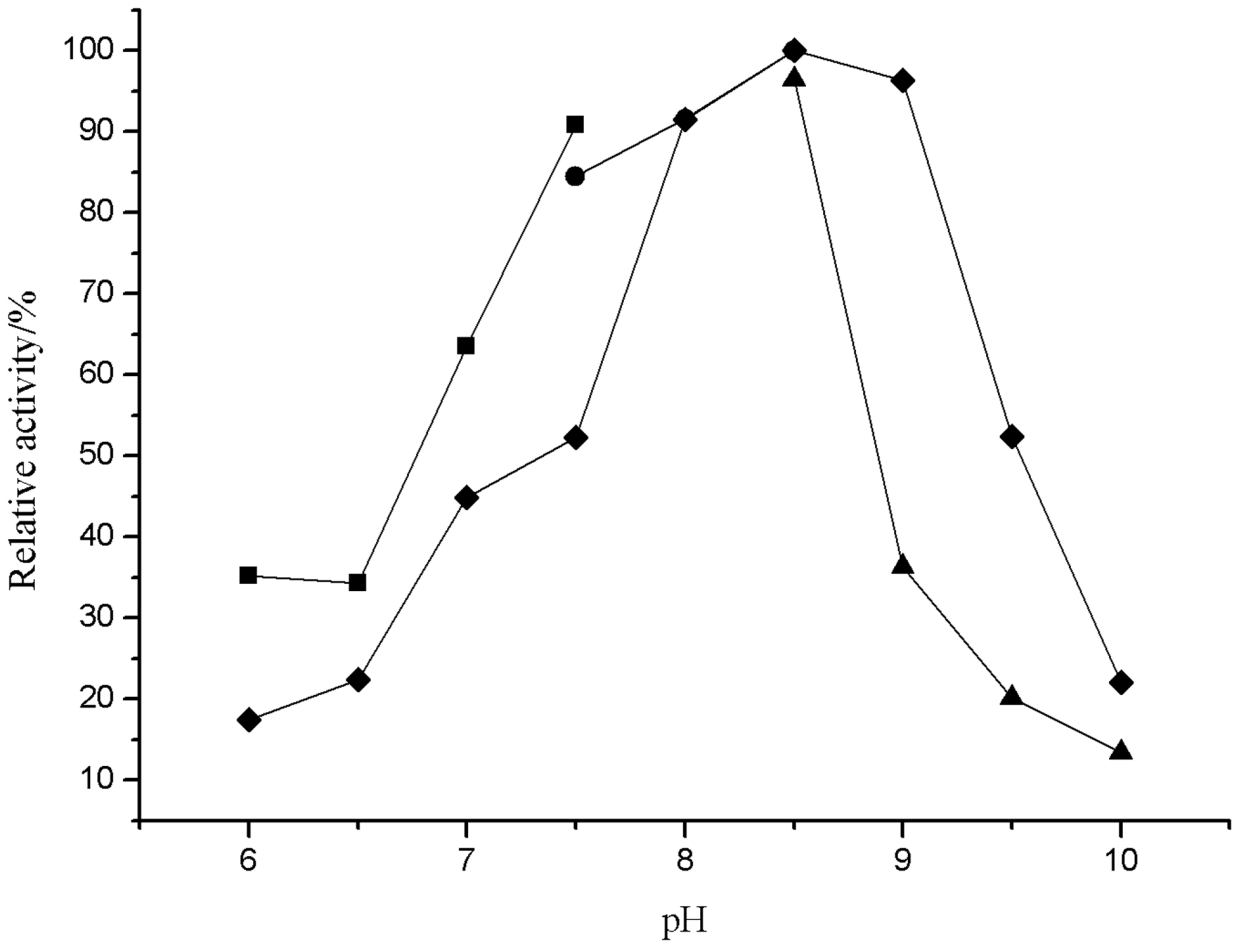
Effect of pH on activity and stabilityof lipase(⧫). For the pH activity: sodium phosphate buffer (▪, pH 6.0–7.5), Tris-HCl buffer (•, pH 7.5–8.5) and sodium carbonate buffer (▴, pH 8.5–10).

The activity of lipase was investigated over a pH range of 6.0–10.0(Fig.4). The results indicated that this enzyme presents an optimal activity at pH 8.5. The enzyme retained more than 63.53% of its activity at pH 7.0, but only 36.36% at pH 9.0. The activity at higher pH values (pH>10) was not tested because of spontaneous hydrolysis of p-NPA [22]. The lipase was stable in the range from pH 8.0 to 9.0.

#### Effect of temperature on enzyme activity and stability

**Figure 5:**
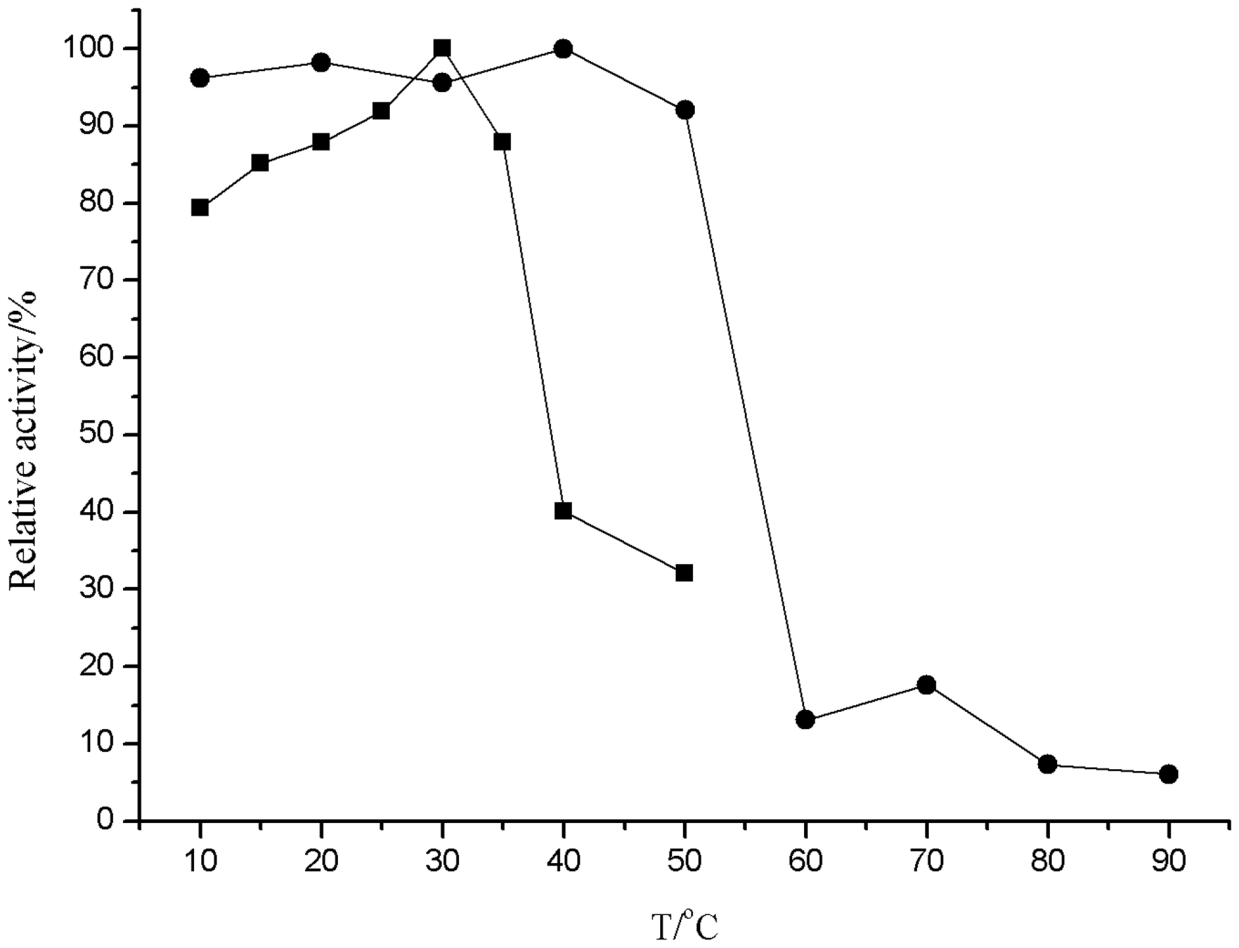
Effect of temperature on activity (▪) and thermal stability (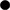) of lipase.

The effect of temperature on lipase activity was studied by assaying the lipase activity at different temperatures. The enzyme was active at a wide range of temperatures (10–35°C), with maximum activity at 30°C (Fig.5).The lipase showed up to 80% of the maximum activity at 10°C. These results indicate the lipase to be a typical cold-adapted Enzyme [23]. In contrast, this enzyme was relatively stable up to 50°C, and maintained about 90% of the maximum activity when incubated for 30 min at 50°C.

#### Effect of various additives on lipase activity

**Figure 6:**
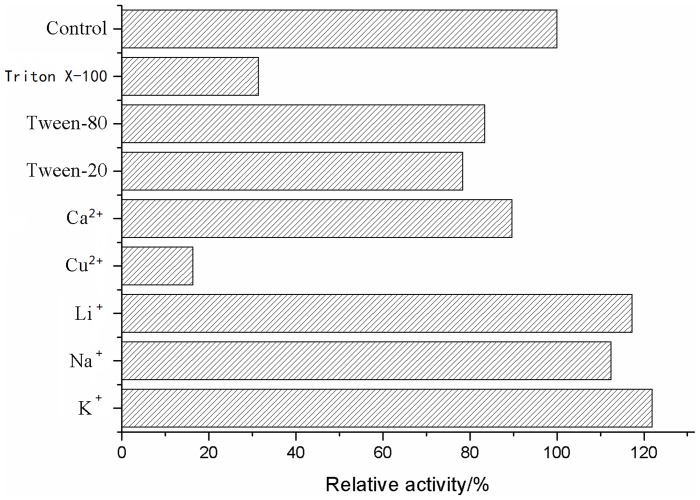
Effects of various metal ions and detergents on lipase activity.

Fig.6 shows the effect of various metal ions and detergents on the lipase activity. Of the cations tested, Ca^2+^ slightly inhibited the activity of the enzyme, whereas the activity was enhanced by Na^+^, Li^+^ and K^+^. Enzyme activity was inhibited by the presence of detergents, such as Tween-20, Tween-80 slightly and Triton X-100.

#### Effect of organic solvents on the lipase activity

**Figure 7:**
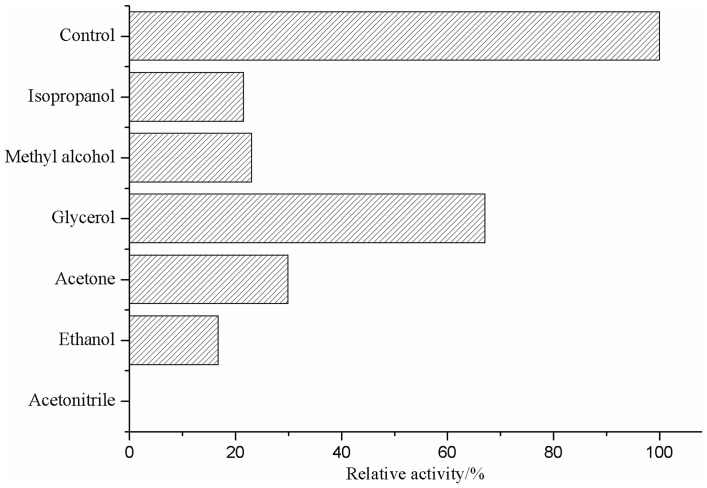
Effect of organic solvents on lipase activity.

As shown in Fig.7, the enzyme was fairly stable in long-chain alcohols but was highly denatured in hydrophilic solvents such as acetone or short-chain (C1–C3) alcohols.

## Discussion

The saline-alkali environment contains a vast pool of novel enzymes and is receiving more and more interest. The halophilic and moderately halophilic bacteria are very hot topics. The halophilic and moderately halophilic bacteria have to face hyperhaline enviroments and have to change the properties of their macromolecules in order to survive. These macromolecules display clearly distinguishable special features such as hyperhaline, organic, hypertonic or extreme temperature tolerance [4], [5].

In recent years, the ability of the halotolerant/halophilic bacteria to grow and produce enzymes over a very wide range of salinities make them very attractive for research and for the isolation of novel enzymes with unusual properties.

We isolated a lipase-producing bacterium, PT-11, from saline soil Shache, Xinjiang Province, northwest of China. Based on morphological, physiological chemotaxonomic and phylogenetic analysis according to the standard method [13], the strain PT-11 is a novel species and is identified as *Oceanobacillus rekensis* PT-11.

Considering different purification processes [24] of lipase, we succeeded in the lipase from Oceanobacillus rekensis PT-11 by Q-Sepharose FF chromatograph and SP-Sepharose FF chromatograph. The molecular mass was estimated to be 23.5 kDa by SDS-PAGE. It was highly active over broad temperature range from 10 to 35°C and show up to 80% of the maximum activity at 10°C.

Typical cold-adapted enzyme [25] take on higher catalytic efficiency at low temperature,have lower optimal reaction temperature,and are more sensitive to heat. Recently, research on cold-adapted lipase has gone down a new direction. Young Ok Kim et al [26] reported cold-adapted lipase of Photobacterium sp. MA1-3 isolated from blood clam and MA1-3 lipase showed optimum activity at 45°C, pH 8.5. Meanwhile, MA1-3 lipase performed a transesterification reaction using olive oil and various alcohols and this lipase produced biodiesel using methanol and plant or waste oils. Florczak T et al [25] also reported a cold-adapted lipase, LipG7, has been purified from the *Antarctic filamentous fungus Geomyces* sp. P7 and LipG7 gone down optimum activity at 35°C, pH 8.0–8.6. Ryu HS et al [27] reported the cold-adapted lipase from *Photobacterium lipolyticum* sp.nov. displayed a maximum activity at 25°C and maintained its activity at a low temperature range (5–25°C).The lipase isolated from *Oceanobacillus rekensis* PT-11 is in accordance with these reports.

The enzyme was found to be cold-adapted, allowing potential application as a biocatalyst in organic chemistry, since psychrophilic enzymes have certain advantages under low-water conditions.

## Supporting Information

Table S1 Utilizing of strains PT-11 to different carbon sources.
(DOCX)

Table S2 Results of API ZYM test of the strains PT-11.
(DOCX)
