# Supplementary material for: Purification and Characterization of a Cold-Adapted Lipase from Oceanobacillus Strain PT-11
Source: PLoS One. 2014 Jul 1;9(7):e101343. doi: 10.1371/journal.pone.0101343 (PMC4077839; doi:10.1371/journal.pone.0101343)
Supplement: Table S1 — Utilizing of strains PT-11 to different carbon sources. (DOCX) [file pone.0101343.s001.docx]

Table S1 Utilizing of strains PT-11 to different carbon sources

| Carbon sources | 1 | 2 |
| --- | --- | --- |
| Dextrin | + | + |
| D-Maltose | + | + |
| D-Trehalose | + | + |
| D-Cellobiose | + | - |
| Gentiobiose | + | - |
| Sucrose | + | + |
| D-Turanose | + | + |
| Stachyose | - | + |
| Vancomycin | + | - |
| Tetrazolium Violet | + | + |
| Tetrazolium Blue | - | - |
| D-Raffinose | - | + |
| α-D-Lactose | - | - |
| D-Melibiose | - | - |
| β-Methyl-D-Glucoside | + | - |
| D-Salicin | + | - |
| N-Acetyl-D-Glucosamine | + | - |
| N-Acetyl-β-D-Mannosamine | + | - |
| N-Acetyl-D-Galactosamine | - | - |
| N-Acetyl-Neuraminic Acid | + | - |
| α-D-Glucose | + | + |
| D-Mannose | + | - |
| D-Fructose | + | + |
| D-Galactose | - | - |
| 3-Methyl-Glucose | - | - |
| D-Fucose | - | - |
| L-Fucose | - | - |
| L-Rhamnose | - | - |
| Inosine | + | - |
| 1% Sodium Lactate | + | + |
| Fusidic Acid | - | - |
| D-Serine | - | + |
| D-Sorbitol | - | - |
| D-Mannitol | + | - |
| D-Arabitol | + | - |
| myo-Inositol | - | - |
| Glycerol | + | - |
| D-Glucose-6-PO_4_ | + | + |
| D-Fructose-6-PO_4_ | + | + |
| D-Aspartic Acid | - | - |
| D-Serine | - | - |
| Troleandomycin | + | - |
| Rifamycin SV | - | - |
| Minocycline | + | + |
| Gelatin | - | - |
| Glycyl-L-Proline | - | - |
| L-Alanine | + | + |
| L-Arginine | - | - |
| L-Aspartic Acid | - | - |
| L-Glutamic Acid | + | + |
| L-Histidine | - | - |
| L-Pyroglutamic Acid | - | - |
| L-Serine | + | + |
| Lincomycin | + | - |
| Guanidine HCl | + | - |
| Niaproof 4 | - | - |
| Pectin | + | + |
| D-Galacturonic Acid | + | - |
| L-Galactonic Acid Lactone | - | - |
| D-Gluconic Acid | + | + |
| D-Glucuronic Acid | + | + |
| Glucuronamide | + | + |
| Mucic Acid | + | - |
| Quinic Acid | - | + |
| D-Saccharic Acid | + | - |
| p-Hydroxy-Phenylacetic Acid | + | - |
| Methyl Pyruvate | + | - |
| D-Lactic Acid Methyl Ester | + | + |
| L-Lactic Acid | + | + |
| Citric Acid | + | - |
| α-Keto-Glutaric Acid | + | - |
| D-Malic Acid | + | - |
| L-Malic Acid | + | + |
| Bromo-Succinic Acid | + | + |
| Nalidixic Acid | + | + |
| Lithium Chloride | + | + |
| Potassium Tellurite | + | - |
| Tween 40 | - | + |
| γ-Amino-Butyric Acid | - | + |
| α-Hydroxy-Butyric Acid | + | + |
| β-Hydroxy-Butyric Acid | + | + |
| α-Keto-Butyric Acid | + | - |
| Acetoacetic Acid | + | + |
| Propionic Acid | + | + |
| Acetic Acid | + | + |
| Formic Acid | - | - |
| Aztreonam | + | + |
| Sodium Butyrate | + | + |
| Sodium Bromate | - | - |

1:*Oceanobacillus profundus* CL-MP28^T^；2:PT-11 “+” Positive，“-” Negative.
