# Supplementary material for: Purification and Characterization of a Cold-Adapted Lipase from Oceanobacillus Strain PT-11
Source: PLoS One. 2014 Jul 1;9(7):e101343. doi: 10.1371/journal.pone.0101343 (PMC4077839; doi:10.1371/journal.pone.0101343)
Supplement: Table S2 — Results of API ZYM test of the strains PT-11. (DOCX) [file pone.0101343.s002.docx]

**Table S2 Results of API ZYM test of the strains PT-11**

| API ZYM | 1 | 2 |
| --- | --- | --- |
| Alkaline phosphatase | + | - |
| Esterase(C_4_) | + | + |
| Esterase lipase(C_8_) | + | + |
| Lipase (C_14_) | - | + |
| Leucine arylamidase | - | - |
| Valine arylamidase | - | - |
| Cystine arylamidase | - | - |
| Trypsin | - | - |
| α-chymotrypsin | - | - |
| Acid phosphatase | - | - |
| Naphthol-AS-B1-phosphohydrolase | + | + |
| α-galactosidase | - | - |
| β-galactosidase | + | - |
| β-glucuronidase | - | + |
| α-glucosidase | + | - |
| β-glucosidase | - | - |
| N-acetyl-β- glucosaminidase | - | - |
| α-mannosidase | - | - |
| α-fucosidase | - | - |

1: *Oceanobacillus profundus* CL-MP28^T^; 2: PT-11. “+” Positive，“-” Negative.
